# The Significance of the Wnt/β-Catenin Pathway and Related Proteins in Gastrointestinal Malignancies

**DOI:** 10.3390/ijms26178130

**Published:** 2025-08-22

**Authors:** Adrianna Romanowicz, Marta Łukaszewicz-Zając

**Affiliations:** 1 Department of Biochemical Diagnostics, Medical University of Bialystok, Waszyngtona 15a, 15-269 Białystok, Poland; adrianna.romanowicz@sd.umb.edu.pl; 2 Department of Neurodegeneration Diagnostics, Medical University of Bialystok, Waszyngtona 15a, 15-269 Bialystok, Poland

**Keywords:** biomarker, gastrointestinal cancer, Wnt/β-Catenin pathway

## Abstract

The Wntβ-catenin signaling pathway is a key regulator of gastrointestinal (GI) tumorigenesis, modulating cellular processes such as proliferation, differentiation, and epithelial-to-mesenchymal transition (EMT). In this review, we evaluate the expression and mutation profiles of core Wntpathway components in the most common GI malignancies. Our findings outline notable alterations in ligands, receptors, co-receptors, and intracellular effectors across different GI cancers. In gastric cancer tissue, elevated levels of Wnt proteins, FZD7 receptor, and LRP5/6, along with β-catenin accumulation and reduced APC expression, are associated with poor prognosis. In colorectal cancer samples, common APC mutations and Wnt ligand overexpression contribute to β-catenin nuclear localization and EMT. Esophageal cancer specimens exhibit co-overexpression of Wnt2 and Wnt5A, as well as receptors such as FZD2 and FZD6, which are linked to worse prognosis and reduced survival. Liver cancer tissue commonly harbors CTNNB1 mutations, which encode β-catenin and are associated with poor differentiation. In pancreatic cancer samples, overexpression of Wnt ligands, FZD receptors, and β-catenin is associated with the presence of distant metastasis and poor clinical outcomes. In conclusion, this pathway represents a promising avenue for identifying novel diagnostic, prognostic, and therapeutic biomarkers in GI cancers, warranting further clinical investigation.

## 1. Introduction

Gastrointestinal (GI) cancers represent a significant global health burden, with high incidence and mortality rates. Among them, colorectal cancer (CRC) is the most frequently diagnosed malignancy within this group and ranks as the fourth most common cancer worldwide, according to GLOBOCAN data [1]. Despite advances in screening programs and treatment strategies, it remains a leading cause of cancer-related mortality, placing fifth among all cancers globally. While CRC dominates in terms of incidence, other GI malignancies, such as gastric cancer (GC), liver cancer (LC), esophageal cancer (EC), and pancreatic cancer (PC), contribute significantly to cancer-related deaths. In 2022, GI cancers were responsible for around 4.8 million new cases and 3.2 million deaths globally, making up over a quarter of all cancer diagnoses and more than a third of deaths attributable to cancer worldwide [1]. Although GI cancers share certain risk factors, their distinct etiologies and epidemiological profiles are key drivers of their development. These differences contribute to significant variations in incidence, prognosis, and therapeutic strategies, highlighting the complexity of diagnosing and managing these malignancies [2,3,4,5,6].

Chronic infections and related conditions play a critical role in carcinogenesis. Helicobacter pylori is a well-established risk factor for GC, while hepatitis B and C viruses contribute to hepatocellular carcinoma (HCC) by promoting chronic inflammation and fibrosis [7,8]. Similarly, gastroesophageal reflux disease (GERD) has been associated with an increased risk of EC [9]. In addition to these factors, smoking remains a major contributor to GI malignancies, significantly increasing mortality rates from both PC and EC [10,11]. Many of these risk factors, including tobacco, alcohol, obesity, lack of physical activity, and metabolic disorders, are preventable. Adopting a healthy lifestyle, which includes regular physical activity, maintaining a healthy weight, and following a balanced diet, is crucial in reducing the burden of GI cancers both now and in the future [12].

A primary challenge is the difficulty of detecting GI malignancies at an early stage. As a result, when these cancers are diagnosed, the options for curative treatment are often severely limited or completely absent. Moreover, late-stage diagnosis of these cancers remains a major barrier to improving survival rates and treatment effectiveness [13]. While imaging techniques such as ultrasound and computed tomography, along with endoscopic examinations such as gastroscopy and colonoscopy, play a role in detecting these diseases, biopsy continues to be the most critical diagnostic tool [14]. These methods must be complemented by effective biochemical diagnostics, including traditional biomarkers, such as CEA (carcino-embyrional antigen) and CA 19-9 (carbohydrate antigen 19-9), as well as emerging biomarkers, which could enhance early detection and improve patient prognosis. In symptomatic patients, biomarkers not only aid in distinguishing malignant tumors from benign conditions but are also important in guiding treatment decisions [15]. Their significance extends beyond diagnosis, as they serve as key indicators in monitoring disease progression and treatment effectiveness. In patients undergoing surgical tumor resection, biomarkers help evaluate the success of the procedure by detecting any remaining cancer cells and identifying early signs of recurrence [16]. Therefore, the main goal of modern medicine is to discover new biomarkers that will be able to detect these deadly malignant tumors in the early stages of the disease with high sensitivity and specificity, which will lead to faster implementation of effective treatment, thus contributing to the longer survival of patients with these tumors. Some clinical investigations have reported that dysregulation of the Wnt pathway, as a critical cell-to-cell communication network, is frequently observed in a wide range of malignancies, promoting tumor initiation, progression, and metastasis. Therefore, the aim of our study is to analyze the current body of research on the proteins associated with the Wnt/β-catenin signaling pathway, specifically exploring their potential utility as novel biomarkers in GI cancer. Moreover, Wnt pathway components have emerged as promising therapeutic targets, with their modulation using inhibitors or activators, supporting the development of new cancer therapies, including GI patients, as presented in our review.

## 2. Methods

A systematic approach was employed to identify, select, and analyze publications related to the Wnt signaling pathway and its components in gastrointestinal cancers. Literature searches were conducted in databases, including PubMed, using relevant keywords. Inclusion and exclusion criteria were applied to select the most relevant and recent studies. The analysis focused on the expression/levels of specific Wnt pathway components in cancer tissues, their potential as biomarkers, and possible clinical implications.

## 3. Wnt/β-Catenin Pathway: General Information

The Wnt signaling pathway consists of both canonical and non-canonical pathways. Non-canonical pathways, such as the Wnt/Ca^2+^ pathway and the planar cell polarity pathway, function independently of the β-catenin-T-cell factor/lymphoid enhancer-binding factor (TCF/LEF) complex [17]. In contrast, the canonical Wnt pathway, also known as the Wnt/β-catenin pathway, involves the translocation of β-catenin into the nucleus, where it activates specific target genes through TCF/LEF transcription factors. The primary role of the canonical pathway is to regulate cell proliferation, whereas non-canonical pathways control cell polarity and migration. These pathways interact and mutually regulate each other.

**Figure 1:**
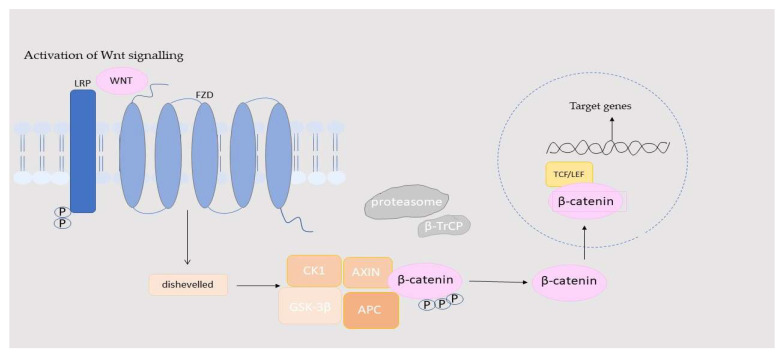
Activation of the Wnt/β-catenin pathway. APC: adenomatous polyposis coli; Axin: Axin proteins; β-TrCP: beta-Transducin Repeat-Containing Protein; CK1: casein kinase I; FZD: specific sevenfold transmembrane receptor Frizzled protein; GSK-3β: glycogen synthase kinase-3β; LRP: phosphorylated lipoprotein receptor; TCF/LEF: β-catenin-T-cell factor/lymphoid enhancer-binding factor.

**Figure 2:**
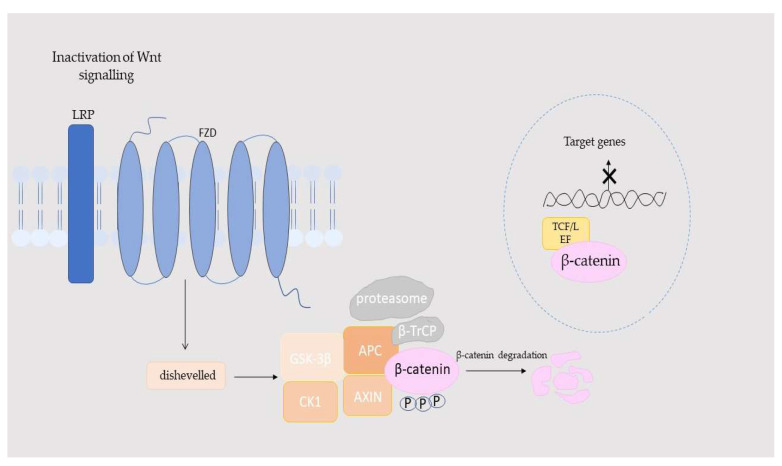
Inactivation of the Wnt/β-catenin pathway. APC: adenomatous polyposis coli; Axin: Axin proteins; β-TrCP: beta-Transducin Repeat-Containing Protein; CK1: casein kinase I; FZD: specific sevenfold transmembrane receptor Frizzled protein; GSK-3β: glycogen synthase kinase-3β; LRP: phosphorylated lipoprotein receptor; TCF/LEF: β-catenin-T-cell factor/lymphoid enhancer-binding factor.

Wnt signaling is involved in the development and renewal of the small intestinal epithelium, promoting the differentiation of Paneth cells in intestinal crypts. Additionally, Wnt signaling is closely linked to liver metabolism and regeneration, lung tissue repair and function, hair follicle renewal, hematopoietic system development, and the maturation and activity of osteoblasts [18,19,20]. The Wnt/β-catenin pathway is composed of four key parts: extracellular signals, membrane receptors, cytoplasmic components, and nuclear elements. The extracellular signals are primarily mediated by Wnt proteins, such as Wnt3A, Wnt1, and Wnt5A. At the membrane level, the main receptors involved are FZD and LRP5/6. The cytoplasmic segment includes proteins such as β-catenin, disheveled (DVL), glycogen synthase kinase-3β (GSK-3β), Axin, adenomatous polyposis coli (APC), and casein kinase I (CK1), which regulate signal transmission. In addition, WLS/Gpr177 (protein Wntless/G protein-coupled receptor 177) is a newly identified chaperone protein that specifically escorts Wnt ligands for secretion. In the nucleus, β-catenin interacts with TCF/LEF transcription factors to activate target genes, such as matrix metalloproteinases protein (MMPs) and c-Myc. This pathway is highly conserved and triggered when Wnt ligands bind to membrane receptors through autocrine or paracrine signaling. Activation stabilizes β-catenin, allowing it to move into the nucleus, where it promotes the expression of genes responsible for cell proliferation, survival, differentiation, and migration [21,22]. When Wnt signals are not present, the receptors FZD and LRP5/6 are located separately on the plasma membrane. In the cytoplasm, a “destruction complex” consisting of proteins such as APC, Axin, CK1, and GSK3 interacts with β-catenin, leading to its phosphorylation and subsequent degradation. This results in Groucho binding to TCF/LEF, which prevents the transcription of certain target genes. However, when Wnt signals are detected by FZD and LRP5/6, the destruction complex is recruited to the membrane, where it interacts with FZD. This disrupts the degradation of β-catenin, allowing it to stabilize and move into the nucleus. Once in the nucleus, β-catenin activates the transcription of target genes through its interaction with TCF/LEF. The movement of β-catenin between the cytoplasm and nucleus is a crucial feature of the activation of the Wnt/β-catenin pathway. Although there are Wnt ligands and FZD receptors in humans, their interactions are only partially promiscuous. For example, Wnt3A can bind and activate the canonical β-catenin pathway via multiple FZDs (such as FZD1, FZD2, FZD4, FZD5, FZD7, FZD8, FZD9, and FZD10), particularly relying on co-receptor LRP6, whereas non-canonical ligands, such as Wnt5A and Wnt4, exhibit more specific interactions with certain FZDs and often signal through non-canonical routes, such as the PCP or Wnt/Ca^2+^ pathways. This selectivity is influenced by structural differences in the FZD cysteine-rich domain and linker regions, which modulate downstream signaling outcomes [23]. These processes are illustrated in Figure 1 and Figure 2.

### 3.1. Elements of the Wnt/β-Catenin Signaling Pathway and Their Roles

The Wnt/β-catenin signaling pathway is a complex mechanism regulating numerous cellular processes. This pathway relies on several key proteins, including FZD, LRP5/6, R-spondins, and their receptors—LGR5, DvL, Axin, APC, GSK-3β, CK-1α, β-catenin, and TCF/LEF [24]. Moreover, DKK1 (Dickkopf-related protein 1), RAC1 (Ras-related C3 botulinum toxin substrate 1), WIF1 (Wnt inhibitory factor 1), SFRP1 (Secreted Frizzled-Related Protein 1), and FRAT1 also interplay with Wnt signaling and are involved in carcinogenesis. DKK1 forms a complex with receptors such as LRP5/6 and Kremen (Kringle Containing Transmembrane Protein 1), causing the internalization and removal of LRP5/6 from the cell surface, so this protein is a secreted inhibitor of Wnt signaling. It was proven that dysregulation of DKK1 and the Wnt pathway is linked to a wide range of diseases, including cancer, promoting tumor cell proliferation, migration, and metastasis [25]. In addition, the inhibitory effect of DKK1 on Wnt signaling makes it a potential therapeutic target for various diseases, including cancer [26]. Rac1 is a small GTPase protein that plays a role in cell adhesion, migration, and proliferation; therefore, Rac1 mutations and aberrant activity are linked to cancer development. Rac1 acts as a modulator of canonical Wnt signaling by increasing this pathway via impacting β-catenin’s nuclear localization and its ability to form β-catenin-LEF-1 complexes, enhancing gene transcription. Some clinical investigations have reported that Rac1 is overexpressed in cancer cells and is implicated in tumor growth, metastasis, and drug resistance [27]. WIF1 inhibits Wnt signaling via direct ligand binding, interacting with Wnt receptors that block Wnt signaling, and promoting the degradation of β-catenin, which inhibits Wnt signaling. It was proven that the loss of WIF1 function can lead to uncontrolled Wnt signaling, promoting tumor growth and metastasis [28,29]. Other proteins that could be regulators of the Wnt signaling pathway are SFRP1 and FRAT1; however, they have opposing effects. SFRP1 functions as an inhibitor of Wnt signaling by binding to Wnt ligands and preventing them from interacting with Frizzled receptors and blocking the canonical Wnt/β-catenin pathway, while FRAT1 interacts with DVL, enhancing canonical Wnt signaling, and thus promoting β-catenin stabilization and nuclear translocation. In addition, overexpression of FRAT1 can contribute to tumor growth and metastasis by enhancing Wnt signaling [30,31,32]. A detailed overview of the key components involved in the Wnt/β-catenin signaling pathway, including their cellular localization, function, and role in either activating or inhibiting the pathway, is provided in Table 1.

### 3.2. The Role of Wnt/β-Catenin Signaling Pathway in Carcinogenesis

The Wnt/β-catenin signaling pathway is important in cancer development, and its dysregulation is often caused by mutations in key components. The Wnt gene was first identified in 1982 as an oncogene in mouse breast cancer [39]. Mutations in tumor suppressors, such as APC, Axin1/2, and RNF43, as well as β-catenin mutations, are common in many cancers [40,41,42,43,44]. Among them, β-catenin mutations are the most frequent. APC, as a part of a cytoplasmic complex, promotes the destruction of the transcriptional licensing factor β-catenin. APC loss stabilizes β-catenin and constitutively activates the pathway even in the absence of a Wnt signal. The mutation of APC eradicates this function, triggering constitutive activation of the canonical Wnt signaling pathway. This process is characteristic of most CRCs. By negatively regulating canonical Wnt signaling, APC is able to promote differentiation, counteract proliferation, facilitate apoptosis, and suppress invasion and tumor progression [45]. APC mutations are frequently found in colorectal adenomas, which are precancerous growths. Furthermore, APC also suppresses the progression and initiation of tumors in the colorectal epithelium through functions that are independent of canonical Wnt signaling [45]. APC mutations, first linked to familial adenomatous polyposis (FAP), are found in 80% of colorectal adenomas and cancers [46,47]. These mutations prevent the formation of the degradation complex, leading to uncontrolled Wnt/β-catenin activation. This process promotes tumor growth, enhances cancer cell self-renewal, and contributes to drug resistance. R-spondins (RSPOs), as a family of secreted proteins, are modulators of the Wnt signaling pathway. Their key receptor is the G protein-coupled receptor LGR5. When R-spondins bind to LGR5, they enhance Wnt signaling, leading to cell proliferation and survival [48]. Cancer stem cells (CSCs) are involved in tumor initiation, growth, drug resistance, recurrence, and metastasis after treatment due to their capacity for self-renewal and differentiation [49]. The maintenance of this ability is regulated by signaling pathways, such as Wnt/β-catenin, transforming growth factor-β (TGF-β), Hedgehog, and Notch. Among these, the significance of Wnt/β-catenin signaling in CSCs is widely recognized. Wnt10B, a member of the Wnt ligand gene family, encodes a secreted protein that specifically activates the highly conserved Wnt signaling cascade [50]. Several signaling pathways, including TGF-β, Wnt, Notch, and PI3K/Akt, which are crucial in tumor growth and progression and are able to interact with each other in complex mechanisms, can either promote or inhibit cancer development depending on the context and tumor stage. Furthermore, these pathways play a significant role in therapy resistance, as cancer cells can adapt to targeted therapies by activating alternative pathways [51]. The TGF-β pathway is characterized as a tumor suppressor, leading to reduced cell proliferation and stimulated apoptosis. However, a more advanced stage of tumor can promote tumor progression, metastasis, and angiogenesis by inducing EMT. The TGF-β pathway can crosstalk with other pathways, such as PI3K/Akt via Akt activation, contributing to cell migration, EMT, and cell survival; EGFR/IL-6R/STAT3, via indirect activation of STAT3 through EGFR and IL-6R signaling, promotes EMT. It was also reported that the Notch pathway can also interact with TGF-β, Wnt, and other pathways to regulate tumor progression [51].

Although activating mutations lead to β-catenin accumulation, heterogeneous nuclear localization of β-catenin within tumor regions and at the invasive front indicates that Wnt ligand overexpression further reinforces pathway activity in specific cellular contexts (e.g., leading to epithelial–mesenchymal transition and invasion) [52]. Wnt signaling maintains CSC properties and contributes to their self-renewal, survival, and resistance to therapy [53,54,55]. Elevated Wnt activity promotes CSC-driven tumor progression, EMT, and the colonization of distant organs [56]. In the tumor microenvironment, stromal cells, such as cancer-associated fibroblasts (CAFs), actively secrete Wnt2, which promotes the migration and invasion of CRC cells [57]. Additionally, fibroblast-produced RSPO3 enhances Wnt signaling and supports tumor growth through synergistic activity [58]. Tumor cells can modulate nearby fibroblasts via PLD2 secretion, inducing a senescence-associated secretory phenotype (SASP) that reinforces Wnt-driven CSC maintenance [59]. Wnt signaling also crosstalks with immune components. Tumor-associated macrophages (TAMs) promote β-catenin pathway activation in CRC cells by upregulating TRIB3, supporting stemness and invasiveness [60]. Furthermore, Wnt signaling affects immune evasion by inducing immune tolerance and limiting anti-tumor immune responses, shaping the immunosuppressive tumor microenvironment [61]. Importantly, overexpression of specific Wnt ligands, such as Wnt10B, can activate the canonical β-catenin/TCF/LEF transcriptional program, reinforcing CSC traits and metastatic capacity [50]. Despite constitutive pathway activation due to mutations, these extrinsic sources of Wnt ligands, produced by both cancer and stromal cells, enhance tumor heterogeneity and functional plasticity through both autocrine and paracrine mechanisms. Additionally, non-canonical Wnt pathways (e.g., Wnt/PCP, Wnt/Ca^2+^) are involved in cytoskeletal remodeling, cell migration, and interaction with growth factors and ECM components in the tumor microenvironment [62,63]. These alternative pathways contribute to invasion, therapy resistance, and stromal remodeling beyond the canonical β-catenin axis.

DKK1, as a critical component in the canonical Wnt pathway, is linked to a variety of GI cancers via its proapoptotic effect on tumor cells, leading to downregulation of β-catenin [26]. Some clinical investigations have reported elevated serum DKK1 levels in ESCC, GC, and PC cancers, indicating its role as a promising biomarker in GI malignancies [64,65,66]. On the other hand, DKK1 expression was reduced in CRC, and it downregulated the expression of VEGF. It may also be involved in the repression of tumorigenesis and angiogenesis in CRC [67]. These discrepancies in the functions of DKK1 in GI cancers can be explained by variations in the interplay of existing signaling pathways, cellular context, and surrounding TME [26].

### 3.3. Wnt Pathway-Targeted Therapy in Gastrointestinal Cancers

A potential therapeutic strategy for GI cancers is β-catenin/TCF disruptors, especially in those with aberrant activation of the Wnt/β-catenin signaling pathway, which may offer a way to inhibit tumor growth and improve treatment response. The Wnt/β-catenin pathway is often abnormally activated in GI cancers, especially in CRC and GC, resulting in mutations in genes such as APC or β-catenin, leading to increased β-catenin levels and its translocation into the nucleus, where β-catenin binds to TCF/LEF transcription factors, activating the transcription of genes involved in cell proliferation, survival, and metastasis. Therefore, targeting this pathway with inhibitors is a promising treatment strategy in GI cancers. Several approaches are under intensive examination to disrupt the β-catenin/TCF interaction and signaling, such as small molecule inhibitors that directly interfere with the binding of β-catenin to TCF or promote β-catenin degradation (XAV939—which stabilizes Axin by inhibiting tankyrase enzymes, as well as PKF118-310—which disrupts the β-catenin/TCF interaction) [68].

Another group of potential anti-cancer agents are natural product-based inhibitors that can inhibit β-catenin signaling via downregulating β-catenin expression, modulating phosphorylation, or promoting degradation [69]. Gene therapy approaches using recombinant adenoviruses carrying a lethal gene under the control of a β-catenin/TCF-responsive promoter are able to selectively target and destroy cancer cells with an active β-catenin pathway [70]. Moreover, β-catenin/TCF disruptor therapy might also be combined with conventional chemotherapeutic agents or other targeted therapies, which may improve the effectiveness and reduce toxicity. For example, the study by Pramanik et al. suggests that capsaicin can reduce β-catenin signaling due to the dissociation of β-catenin/TCF-1 complex, which may lead to apoptosis and suppression of tumor growth in PC [70,71].

Ligand-blocking antibodies prove to be significant in GI immunotherapy due to their function in targeting and blocking the interaction between ligands and their receptors on tumor or immune cells. This process strengthens the immune response against neoplasms and specifically targets immune checkpoint proteins such as PD-1 and its ligands (PD-L1 and PD-L2), as well as other targets such as CTLA-4, to enhance the anti-tumor effects of T cells, preventing them from attacking cancer cells [72]. For example, antibodies such as nivolumab (anti-PD-1) and pembrolizumab (anti-PD-1) have been reported to be promising agents in the treatment strategy of various GI cancers, including GC and CRC.

Small molecules and biologics targeting the Wnt signaling pathway are being explored as novel, potential therapies for GI cancers, especially CRC, GC, and PC. These include target agents such as the Wnt ligand inhibitors Ipafricep and LGK794, and the Wnt receptor/co-receptor inhibitors Rosmantuzumab and Vantictumab. Small molecules inhibit β-catenin activity and downstream gene expression, causing cell cycle arrest and apoptosis; e.g., C644-0303 stabilizes the destruction complex, while AZ1366 and Hydroxychloroquine are able to compound and disrupt the β-catenin/TCF complex. It was shown that the presented therapies aim to inhibit the overactive Wnt signaling pathway, which is frequently implicated in GI cancer development and progression [73,74,75].

## 4. The Role of the Wnt/β-Catenin Pathway and Its Components as Potential Biomarkers in Gastrointestinal Cancers

### 4.1. Gastric Cancer

#### 4.1.1. Ligands, Receptors, and Co-Receptors

The homeostasis of the gastrointestinal epithelium depends heavily on a fine balance between cell proliferation, cell cycle arrest, and migration. In addition to its essential role in embryonic development, the canonical Wnt signaling pathway is critical for regulating cell proliferation, maintaining stem cell compartments, and preserving homeostasis in the normal gastric mucosa [44,76,77,78]. Conversely, it is well established that disruptions in the Wnt pathway are closely linked to oncogenesis, with aberrant activation of the Wnt/β-catenin signaling pathway observed in more than 30% of GC cases. In this context, various components of the Wnt pathway may be overexpressed or activated through alternative mechanisms [79,80,81,82].

Among the 19 identified Wnt ligands, many display altered expression patterns in GC, influencing tumor progression and treatment response. For instance, Wnt2 is selectively overexpressed in GC tissues and is significantly associated with advanced disease stages (III and IV) compared to early stages (I and II; *p* < 0.001), as well as the presence of lymph node metastasis (N1) in comparison to the N0 subgroup (*p* < 0.001) [83]. In vitro studies have shown that Wnt2 promotes cancer cell migration and invasion, reinforcing its role in disease progression. Similarly, Wnt3 is markedly upregulated in gastric tumor tissues, where it activates oncogenic signaling through the β-catenin pathway. Inhibition of Wnt3 leads to the suppression of β-catenin and cyclin D1 expression, resulting in reduced cell proliferation, impaired migration, and increased apoptosis. Wang et al. found that silencing Wnt3 expression through siRNA technology could represent a novel and effective therapeutic approach [84]. Additionally, Wnt6 expression is linked to advanced tumor stages, lymph node involvement, and a reduced response to ECF chemotherapy (epirubicin, cisplatin, and 5-fluorouracil) [85]. In contrast, Wnt5A primarily engages the non-canonical Wnt/Ca^2+^ pathway, which is implicated in EMT and GC metastasis. Notably, Wnt5A expression is observed in 77.6% of GC cases and correlates with deep tumor invasion, lymph node metastasis, and unfavorable prognosis [86]. Its interaction with FZD2 promotes cancer cell invasion, while suppression of ZEB1, a transcriptional regulator of Wnt5A, has been shown to reduce tumor cell proliferation and enhance apoptosis [87,88]. Following ligand binding, the Wnt signal is transmitted through FZD receptors, which are seven-transmembrane coiled-coil proteins with 10 identified variants (FZD1–FZD10) in humans. These receptors play a dual role in GC cells, contributing to both the promotion and suppression of tumors. High levels of FZD8 expression in GC patients are an indicator of poor prognosis [89]. FZD2 and FZD7 can be used as markers to assess both the treatment response and prognosis of patients, aiding in personalized therapy. The level of FZD2 can predict median overall survival (OS), the degree of immune cell infiltration, and the immune response, while also evaluating the effectiveness of immunotherapy. Elevated expression of FZD7 is linked to an elevated incidence of GC and increased lymph node metastasis [90]. In contrast to other isoforms, FZD5 and FZD6 exhibit anti-oncogenic properties. FZD5 preserves the epithelial phenotype of GC cells, inhibits EMT, and reduces their metastatic potential, while FZD6 suppresses the proliferation and migration of these cells [91,92]. Additionally, the FZD10 protein, found in exosomes derived from plasma, shows a strong correlation with its expression in GC tissues, linking it to advanced disease stages and poor prognosis [93]. This discrepancy in the role of FZD receptors in GC development is a result of complex behavior rooted in their involvement in multiple Wnt signaling pathways, with varying outcomes depending on the specific FZD subtype, context, and signaling branch. Different cell types express different combinations of these receptors, leading to context-dependent signaling outcomes. LRP5 and LRP6 act as key co-receptors in the Wnt signaling pathway. High expression of LRP5 is associated with advanced clinical stages and poor prognosis in tissues from GC patients [94]. In 2022, Zheng et al. discovered that LRP6 interacts with capillary morphology genesis gene 2 (CMG2) to sustain the stemness of GC stem cells and promote the progression of the disease [95]. It was shown that separate binding sites for different subsets of Wnt isoforms can determine the enhancement or reduction in signaling conferred by LRP6 antibodies. The complexity of co-receptor-ligand interactions could provide differential regulation of signaling by Wnt isoforms and can be used with antibodies to differentially manipulate Wnt signaling in specific tissues or disease states [96].

#### 4.1.2. Intracellular Components

As a central component of the Wnt signaling pathway, β-catenin is a significant factor in the development and progression of GC. Its abnormal expression in gastric cancer tissues, detected through immunohistochemistry, has been associated with poor OS and adverse histopathological features, including unfavorable Lauren classification, lymph node and distant metastases, and low tumor differentiation [97]. β-catenin expression was detected in 90.63% of poorly differentiated gastric adenocarcinomas [98]. This percentage is significantly higher than in chronic superficial gastritis and precancerous lesions, suggesting its potential as a prognostic marker of tumor aggressiveness and metastatic behavior. APC, a key negative regulator of the Wnt signaling pathway and a crucial component of the destruction complex, is downregulated in GC due to the overexpression of miRNA-192 and miRNA-215. This suppression leads to increased Wnt activity, promoting cancer cell proliferation and migration [99]. While GSK-3β is often linked to pro-tumorigenic roles in other malignancies, its expression in GC appears to act differently, highlighting the tissue-specific complexity of Wnt signaling. In GC, GSK-3β expression was significantly higher in the early stages of pTNM than in the advanced stages and inversely correlated with lymphatic invasion and metastasis. Moreover, its expression was associated with better prognosis, including longer overall survival, and positively correlated with tumor-suppressor proteins and cell cycle regulators [100]. The conflicting behavior of GSK-3β in GI cancers could be explained by the fact that GSK-3β inhibits the Wnt/β-catenin pathway—a major oncogenic driver in GC—and its expression is often reduced or inactivated in aggressive GC. These contrast with other GI cancers, where GSK-3β promotes the growth of tumors via alternative pathways. Presented findings highlight the tissue-specific complexity of Wnt signaling and the contextual role of GSK-3β in GC. What is crucial is that TME may strongly influence Wnt’s role in GC, because Wnt ligands are secreted by cancer-associated fibroblasts (CAFs) or tumor-associated macrophages (TAMs). In conclusion, canonical Wnt acts oncogenically on GC cells, while non-canonical Wnt signaling has proved to be tumor-suppressive in some model systems. Moreover, the dual role of Wnt in GC can also be explained by variability in ligand type, receptor expression, microenvironment cues, and downstream interactions.

#### 4.1.3. Wnt Pathway-Targeted Therapy in Gastric Cancer (GC)

Many investigators confirm that DKK1 is an ideal anti-cancer therapeutic target [101]. DKK1 promotes an immunosuppressive TME that benefits MDSC expansion by regulating β-catenin-independent Wnt signaling, resulting in suppression of CD45+ T cell proliferation, and contributing to cancer immune evasion [101]. In addition, cancer cells via DKK1 acquire stem cell-like functions by preventing Wnt activation regardless of β-catenin dependence. A new pathway indicates that DKK interacts with CKAP4 receptors, causing activation of Akt signaling and stimulating tumor cell proliferation [101]. It was shown that a monoclonal antibody that specifically targets and neutralizes DKK1 (DKN-01) is being investigated in clinical trials. Studies reported that DKK1 is a potential candidate for treating advanced GC. The therapeutic strategy of evaluating a combination of DKK1 and an immune checkpoint inhibitor is promising for tumor patients with high DKK1 expression. The clinical findings also assessed that in the combination treatment with DKN-1 and pembrolizumab, patients with an elevated DKK1 level in the tumor demonstrated relatively higher efficacy than those with a low level. The same effect was observed in patients who were resistant to PD-1 treatment. Moreover, DKN-01 monotherapy had much less efficacy than combination therapy with an anti-PD-1 antibody; thus, further studies on DKK1 monotherapy are crucial to explore the structural mechanism of DKK1 inhibition of the Wnt pathway [101]. It was proven that the domain of DKK1 can regulate the ability to bind to LRP6. Chi et al. demonstrated two distinct conformations of the DKK1 structure with different interactions of N and C domains; thus, it is important to explain how different configurations of the N-terminal domains of DKK1 affect the Wnt signaling pathway via interaction with the C-terminal domains. This knowledge will be crucial to discover the new targeting domain on DKK1 for further drug development [101].

### 4.2. Colorectal Cancer

#### 4.2.1. Ligands, Receptors, and Co-Receptors

Wnt ligands, along with their receptors and co-receptors, are able to activate the Wnt/β-catenin signaling cascade and promote CRC progression [102]. Expression of Wnt1 is reduced in tumor tissues, which may contribute to the advancement of disease by weakening the inhibition of cancer cell migration and invasion. In contrast, Wnt2 is markedly upregulated and appears to enhance Wnt/β-catenin activity in malignant cells [103]. Likewise, overexpression of Wnt3A further amplifies this signaling, leading to alterations in cell morphology, modulation of EMT-associated proteins, and increased clonogenicity and invasiveness [104]. Elevated levels of Wnt4 have been detected in the serum of patients with CRC [105]. Tissues from CRC patients were recognized as a significant source of increased Wnt4 levels. Notably, serum Wnt4 concentrations decreased following surgical removal of the tumor. These findings suggest that serum Wnt4 may serve as a potential biomarker for CRC. In contrast, Wnt5A expression is frequently diminished in primary colon tumors and negatively correlates with EMT marker levels. Interestingly, although often downregulated, higher Wnt5A expression has been linked to improved prognosis, indicating a possible tumor-suppressive function in certain contexts [106]. This could be explained by the ability of Wnt5A to suppress EMT and inhibit canonical Wnt signaling, which makes it a context-dependent tumor suppressor.

At the receptor level, immunohistochemical (IHC) analysis has revealed significantly increased expression of FZD6 in CRC tumor samples compared to adjacent non-cancerous tissue [107]. FZD7 is also highly expressed, particularly in the central regions of the tumor mass. Notably, its expression declines at the invasive front despite continued Wnt/β-catenin signaling, suggesting that local microenvironmental factors may influence its regulation, e.g., via spatial heterogeneity within tumors, especially between the proliferative core and the invasive front. Cells within TME are able to adapt to microenvironmental signals by modulating receptor expression, even if the downstream Wnt pathway is still needed [108]. Additionally, increased expression of phosphorylated LRP6 (p-LRP6) was significantly associated with deeper tumor invasion (T3/T4) and the presence of lymph node metastasis. Positive p-LRP6 staining correlates with shorter disease-free survival (DFS), supporting its utility as a prognostic biomarker. Its strong association with tumor progression and recurrence highlights p-LRP6 as a candidate marker for poor clinical outcomes of CRC [109].

#### 4.2.2. Intracellular Components

Intracellular components, such as APC, β-catenin, GSK3β, and CK1, are essential regulators of the Wnt/β-catenin signaling pathway. A study analyzing tissue samples from 720 patients demonstrated that the shift in β-catenin localization from the cell membrane to the cytoplasm and nucleus is significantly associated with poor prognosis when OS is used as the outcome measure [110]. β-catenin plays a dual role in cancer biology: it is not only a key effector of the Wnt signaling pathway but also an essential structural component of adherens junctions, where it links E-cadherin to the actin cytoskeleton. Loss of membrane-associated β-catenin at the invasive front, often due to disruption of the E-cadherin/β-catenin complex, results in weakened cell–cell adhesion and promotes EMT and cancer cell invasiveness [111]. The absence of membranous β-catenin is particularly evident at the invasive front of CRC, and both its membrane localization and the invasive front itself serve as predictive markers of prolonged DFS. In contrast, increased nuclear accumulation of β-catenin correlates with reduced DFS, lower OS, and a higher incidence of lymph node metastasis [112]. These findings indicate that β-catenin plays a dual role in cancer biology, including CRC, due to its function in two distinct cellular contexts: as a transcriptional co-activator in the Wnt/β-catenin signaling pathway, and as a cell adhesion molecule in cadherin-based adherens junctions. These two functions of β-catenin are mechanistically and spatially distinct; depending on the cellular context, tumor type, and microenvironment, β-catenin can either promote or restrain tumor progression.

Approximately 70% of both sporadic and hereditary CRC cases harbor deletions or mutations in the APC gene, underscoring its central role in tumor initiation. Loss-of-function mutations in APC, a hallmark of FAP, also frequently occur in sporadic cases. As a core component of the β-catenin destruction complex, APC dysfunction leads to the cytoplasmic accumulation of β-catenin and aberrant activation of the Wnt/β-catenin pathway, thereby promoting uncontrolled cell proliferation, impaired differentiation, and colorectal carcinogenesis [113]. The evaluation of the presence of APC or B-catenin mutations could help to identify individuals at higher risk of developing CRC, especially in the context of adenomas, thus APC mutations in colon stem cells may cause the formation of polyps and are considered initiating events in colorectal tumorigenesis (polyps) [114]. MSI (microsatellite instability status), particularly MSI-H (microsatellite instability–high), has significant implications in CRC diagnosis and therapy. Some authors believe that MSI-H cases are associated with a generally better prognosis compared to MSS (microsatellite stable) cases because MSI-H tumors tend to respond better to certain types of treatment, particularly immunotherapy, and may have a different pattern of recurrence [115]. These results suggest that the assessment of the presence of APC or B-catenin mutations could improve personalized treatment strategies [45,114]. Several studies have shown that both the expression and activity of GSK3β are elevated in CRC. This kinase contributes to enhanced cell proliferation and modulates chemoresistance through the NF-κB signaling pathway [116]. Moreover, mutations in CK1 isoforms have been shown to affect tumor cell growth and proliferation, with in vivo studies demonstrating their role in promoting tumor growth in xenograft models. Elevated CK1 expression levels are also associated with poorer survival outcomes in CRC patients [117].

#### 4.2.3. Wnt Pathway-Targeted Therapy in Colorectal Cancer (CRC)

The most effective treatment strategy in CRC among the many available options includes porcupine and β-catenin [118]. However, Tankyrases (TNKS1 and TNKS2) are a group of enzymes involved in the Wnt signaling pathway that play a crucial role in tumor cell growth and development, including CRC. Mutations in the APC gene, involved in Wnt pathway regulation, are common in CRC. Some clinical investigations showed that tankyrase inhibitors might be promising anti-cancer agents, especially for APC-mutated CRC [68]. However, in preclinical studies, this therapy has been limited by on-target toxicity in the GI tract, such as enteritis or epithelial degeneration [68]. Recently, a new tankyrase-selective inhibitor—STP1002—has been developed and characterized using in vitro and in vivo studies [68]. The authors indicated that STP1002 revealed anti-tumor activity by stabilizing Axins and antagonizing the Wnt/β-catenin pathway in a subset of APC-mutated CRC cell lines. In addition, STP1002 showed no significant on-target toxicity in the GI tract. These findings confirm that STP1002 is a new, orally active tankyrase inhibitor that presents preclinical antitumour efficacy without on-target toxicity in the GI tract. The authors suggest that STP1002 could be a potential tankyrase-targeted drug in patients with APC-mutated CRC, which was confirmed in clinical trials [68].

### 4.3. Esophageal Cancer

#### 4.3.1. Ligands, Receptors, and Co-Receptors

Wnt2, a canonical Wnt ligand, is implicated in esophageal squamous-cell carcinoma (ESCC) carcinogenesis, being absent in normal esophageal epithelium but present in over 50% of ESCC cases, with expression increasing with tumor grade [99]. Its expression positively correlates with that of FZD2, which is upregulated in 69% of primary ESCC tumors and associated with poor prognosis. Functionally, FZD2 promotes ESCC cell migration and invasion through the STAT3 signaling pathway [119]. Wnt3, an activator of Wnt/β-catenin signaling, has also been linked to ESCC progression and poor outcomes [120]. Similarly, Wnt5A promotes lymphatic metastasis and is associated with poor prognosis, likely through induction of EMT via SNAIL upregulation and E-cadherin downregulation. These findings suggest Wnt5A as a potential therapeutic target [121]. Moreover, Wnt6 was found in the plasma of tumor cells, with nearly 50% of patients exhibiting high Wnt6 expression. Its expression was also linked to various clinicopathological features, such as age, gender, tumor stage, and histopathological type. Importantly, Wnt6 was identified as an independent prognostic factor, with elevated levels correlating with shorter OS and DFS in ESCC patients [122]. Cao et al. found that high FZD6 expression was associated with reduced OS and PFS in ESCC patients [123]. Moreover, FZD7 overexpression, poor tumor differentiation, lymph node metastasis, and advanced TNM stage were associated with worse outcomes. Among them, FZD7 expression, poor differentiation, and TNM stage were identified as independent prognostic factors [124]. Finally, LRP6 expression was elevated in ESCC tissues and associated with several clinical features, including poor differentiation and lymph node involvement. Knockdown of LRP6 reduced cell proliferation, and its expression was positively correlated with the oncogene FGF8, suggesting a role in tumor progression [125].

#### 4.3.2. Intracellular Components

Various studies have demonstrated that the Wnt/β-catenin signaling pathway promotes the initiation and progression of ESCC. Axin acts as a negative regulator of this pathway, and genetic alterations in the Axin1 gene have been implicated in the development of various tumors. Li et al. reported reduced Axin expression in 46% of ESCC tumor samples, which was significantly associated with poor prognosis [126]. In a separate study, β-catenin was found to accumulate abnormally in the nucleus of some ESCC patients, suggesting a potential link between its nuclear localization and esophageal tumorigenesis. Notably, this aberrant localization was not associated with mutations in either the Axin1 or CTNNB1 genes, indicating that post-translational or regulatory mechanisms may be involved [127]. Furthermore, lower Axin expression was shown to correlate with the presence of lymph node metastasis, invasion depth, and lymphatic invasion, implying that decreased Axin levels may contribute to ESCC progression [128]. Additionally, Peng et al. observed a gradual decrease in the expression of E-cadherin, APC, and cyclin D1 in ESCC tissues, with a concurrent increase in β-catenin levels as tumors progressed from well- to poorly differentiated grades. Their findings revealed that β-catenin expression was negatively correlated with E-cadherin, positively correlated with cyclin D1, and showed no significant association with APC expression. These results suggest that APC, β-catenin, cyclin D1, and E-cadherin are actively involved in ESCC pathogenesis and may serve as useful biomarkers for the early detection of EC [129]. Moreover, mutations in APC and β-catenin can contribute to EC development and progression by disrupting normal Wnt signaling. This results in the accumulation of β-catenin in the cell’s cytoplasm and nucleus, leading to the activation of downstream genes that promote cell proliferation, survival, and ultimately, tumor development. Identifying these mutations, especially in the context of Barrett’s esophagus as a precancerous condition, could be useful in identifying patients at higher risk of developing EC [130]. In addition, some clinical investigations have reported that certain β-catenin mutations have been associated with tumor aggressiveness and metastasis, potentially aiding in predicting disease progression [131]. Moreover, understanding the roles of these mutations could guide the selection of targeted therapies [30].

#### 4.3.3. Wnt Pathway-Targeted Therapy in Esophageal Cancer (EC)

Some clinical investigations have reported the important role of porcupine (PORCN) and DKK1 as potential therapeutic targets in GI cancer, especially EC and GC [75,132,133]. Porcupine is an enzyme responsible for the palmitoylation of Wnt proteins, which is crucial for their secretion and activation of the Wnt signaling pathway [134]. Preclinical studies have shown that inhibiting PORCN can effectively disrupt Wnt signaling, reducing tumor growth in GI cancers, including EC [135]. It was reported that pharmacological inhibition of the Wnt/β-catenin signaling pathway makes EC cells more susceptible to chemoradiotherapy. Thus, blocking this pathway makes conventional EC treatments, such as chemotherapy and radiation, more effective. Wnt suggested a synergistic effect when combined with porcupine inhibitors [135] in a Phase 1 clinical trial concerning two porcupine inhibitors, LGK974 (Wnt974) and CGX1321, in advanced GI cancers, including EC and GC cancers. LGK974 was evaluated as a monotherapy in Wnt-dependent advanced solid tumors, indicating that it may influence immune cell recruitment to tumors and potentially enhance the efficacy of checkpoint inhibitors [136]. Moreover, the study of combined therapy of LGK974 and the anti-PD-1 monoclonal antibody, spartalizumab (PDR001), along with further immune signature analyses, is also still under investigation [75].

### 4.4. Liver Cancer

#### 4.4.1. Ligands, Receptors, and Co-Receptors

HCC accounts for approximately 90% of all primary liver malignancies. The elevated expression of Wnt3a in HCC has been linked to poor tumor differentiation, liver cirrhosis, hepatitis B virus (HBV) infection, advanced TNM stage, and reduced OS [137]. Similarly, Wnt1 is overexpressed in human HCC tissues and hepatoma cell lines, with its increased levels correlating with a higher risk of tumor recurrence following curative resection [138]. In addition to Wnt3A and Wnt1, upregulation of Wnt3A, Wnt4, Wnt5A, and Wnt10B has also been observed in both tumor tissue and the adjacent non-tumorous liver [139]. FZD receptors, which serve as key components of Wnt signaling, have also been implicated in HCC progression. Among them, FZD2 has been shown to promote cell migration, invasion, and EMT, supporting its oncogenic role in LC. High FZD2 expression has been associated with poorly differentiated, advanced-stage tumors and reduced recurrence-free survival in HCC patients [140]. FZD7, the most extensively studied Frizzled receptor in HCC, is upregulated in both tumor areas and surrounding dysplastic tissue. Its deregulation appears to be an early event in hepatocarcinogenesis, indicating a potential role in the initial stages of tumor development. Increased FZD7 expression has been linked to the nuclear accumulation of wild-type β-catenin and subsequent activation of the Wnt/β-catenin pathway, reinforcing its contribution to HCC progression [141]. Notably, Wnt3 has been shown to activate this signaling cascade through direct interaction with FZD7 [141]. Moreover, 45–75% of HCC tumor samples reveal elevated LRP6 expression compared to normal liver tissue. This increased expression is linked to tumor aggressiveness, poor prognosis, and resistance to chemotherapy [142].

#### 4.4.2. Intracellular Components

β-catenin expression has been detected in 78% of HCC cases, predominantly at the cell membrane, sometimes accompanied by cytoplasmic and/or nuclear localization. In contrast, normal hepatocytes do not express β-catenin. Membranous and cytoplasmic expression is more frequently observed in well- and moderately differentiated tumors, while nuclear accumulation is more common in poorly differentiated HCC, suggesting a correlation between subcellular localization and tumor grade. Moreover, nuclear β-catenin is more frequently observed in HBV-related HCC (38%) than in hepatitis C virus (HCV)-related cases (15%), and is absent in tumors associated with non-B and non-C hepatitis [143]. Aberrant activation of the Wnt/β-catenin signaling pathway is a key driver of hepatocarcinogenesis and is frequently observed in HCC patients. The most prevalent mechanism involves mutations in exon 3 of the CTNNB1 gene, which encodes β-catenin, found in approximately 20–40% of HCC cases. These mutations are among the most frequent genetic alterations in liver cancer and occur more often in HCV-related HCC compared to HBV-related tumors [144,145]. In addition, inactivating mutations in components of the β-catenin destruction complex, such as Axin1, Axin2, GSK-3β, and APC, can also lead to pathway activation. Axin1 mutations are reported in 3–16% of HCC cases, while Axin2 mutations occur in approximately 3% [146]. Elevated levels of phosphorylated GSK-3β and overexpressed β-catenin are also commonly observed in HCC tissues, even in the absence of CTNNB1 mutations [147].

#### 4.4.3. Wnt Pathway-Targeted Therapy in Liver Cancer

The preclinical study has indicated that PRI-724 is able to suppress the interaction between β-catenin and its co-activator CREC-binding protein (CBP), resulting in inhibition of proliferation and increased apoptosis in β-catenin-activated HCC [148]. It was shown that Tegavivint (BC2059) is a novel inhibitor that interferes with β-catenin and transducin β-like protein 1 (TBL1) interaction and reduces the ability of tumor cells to proliferate. Therefore, it could be a promising anti-tumor in HCC [131,149].

### 4.5. Pancreatic Cancer

#### 4.5.1. Ligands, Receptors, and Co-Receptors

Pancreatic ductal adenocarcinoma (PDAC) is a highly aggressive and treatment-resistant malignancy, with a 5-year OS rate of just 10%. Among the factors contributing to PDAC progression, Wnt2, secreted by activated pancreatic stellate cells, plays a pivotal role by activating the Wnt/β-catenin signaling pathway. Its elevated expression correlates with increased cell migration, invasion, and metastasis and is significantly associated with poor clinical outcomes in PDAC patients [150]. No significant correlation was found between Wnt protein expression and the initial tumor stage or lymph node involvement, although Wnt3A expression was associated with the development of distant organ metastasis following curative surgery (*p* = 0.029) during the follow-up period [151]. In well-differentiated pancreatic carcinomas, Wnt5A exhibits strong immunoreactivity at the luminal surface of glandular structures. In contrast, its expression is markedly reduced in poorly differentiated tumors. The authors believe that it could be explained by the differentiation-dependent role of Wnt5A in maintaining epithelial architecture and restraining tumor aggressiveness, suggesting a potential role in tumor differentiation [152]. Moreover, Wnt7A expression is significantly higher in PC tissues compared to adjacent non-tumorous tissues. This increase is positively associated with poor prognosis and the presence of lymph node metastasis [153]. FZD receptors, elevated protein expressions of FZD1, FZD2, FZD3, FZD4, FZD5, FZD6, FZD7, FZD8, and FZD10, have been observed in PDAC tissues versus adjacent non-cancerous tissues. Recent bioinformatic analyses have shown that members of the FZD gene family are dysregulated in pancreatic adenocarcinoma (PAAD). Specifically, the FZD1, FZD2, FZD6, FZD7, and FZD8 genes were found to be significantly overexpressed in tumor tissues compared to normal pancreatic tissues, based on mRNA expression data from TCGA and other public databases. High FZD2 and FZD7 gene expression correlated with more advanced clinical stages, while FZD3 and FZD6 were identified as independent prognostic markers of PC patients’ survival. These findings suggest that certain FZD genes may serve as potential biomarkers of tumor progression and therapeutic targets in PAAD by modulating the Wnt/β-catenin signaling pathway [154].

#### 4.5.2. Intracellular Components

In pancreatic tumors and adjacent normal tissues, the expressions of key proteins involved in β-catenin degradation, including GSK3β, Axin, and APC, vary significantly. GSK3β levels are consistently higher in tumor samples compared to normal tissue. Axin expression remains mostly unchanged, although a subset of tumors shows increased Axin levels, which are consistently associated with high β-catenin. APC expression is similar between tumor and non-tumorous tissues. These findings suggest that, despite the upregulation of GSK3β—potentially as a compensatory or unrelated response—the lack of corresponding increases in Axin and APC may impair β-catenin degradation, thus contributing to its accumulation in PC [155]. Additionally, high expressions of PROX1 and β-catenin are frequently observed in pancreatic tumors. Elevated β-catenin levels are notably associated with a lower tumor grade. When both PROX1 and β-catenin are highly expressed, they are independently linked to improved survival in PDAC, and their combined high expression further strengthens this association [156].

#### 4.5.3. Wnt Pathway-Targeted Therapy in Pancreatic Cancer (PC)

A growing body of evidence reports that in cancer models, certain GSK3β inhibitors can improve tumor sensitivity to chemotherapeutic agents [157,158,159]. The pharmacologic inhibition of GSK3β with 9-ING-41(Elraglusib) can disrupt gemcitabine-induced DNA repair processes, leading to stimulated apoptosis in chemoresistant PC cells, which was reported in vitro and in vivo studies [160]. Moreover, the Phase 2 study is still being evaluated to assess the efficacy of combining FOLFIRINOX (Folinic Acid, 5-Fluorouracil, Irinotecan, and Oxaliplatin) with 9-ING-41, a GSK-3β inhibitor, and Losartan, a TGF-β blocker, in treating adults with untreated metastatic pancreatic adenocarcinoma. In addition, GSK3β inhibitor, LY2090314, has been reported to modulate the intrinsic PC cell resistance with chemotherapeutic agents, such as Oxaliplatin and gemcitabine [161]. Furthermore, preclinical investigations have also demonstrated that drug combinations with LY2090314 decrease PC cell viability, whereas mouse I confirm models treated with LY2090314 and nab-paclitaxel exhibited improved overall survival and reduced cytotoxicity in non-malignant pancreatic cells [13,157,158,159,160,161].

Table 2 provides a concise summary of selected components of the Wnt/β-catenin signaling pathway, highlighting their clinical significance and relevance as diagnostic, prognostic, or therapeutic biomarkers.

## 5. Perspectives and Clinical Implications

The goal of future medicine is to develop more personalized and effective treatment strategies for patients with GI malignancies, with the aim of improving patient outcomes and survival of patients with these malignancies. In GI, aberrant activation of the Wnt/β-catenin signaling pathway is frequently observed and contributes to uncontrolled cell proliferation, tumor progression, and therapeutic resistance. Components of this pathway, including Wnt ligands (e.g., Wnt1, Wnt2B, Wnt3, Wnt5A, Wnt7B, and Wnt10A), Frizzled receptors (such as FZD2, FZD7, FZD8, and FZD9), downstream effectors such as β-catenin and TCF/LEF transcription factors, and inhibitors such as DKK1, are increasingly recognized as promising diagnostic and prognostic biomarkers in colorectal, gastric, pancreatic, and esophageal cancers. These molecules also hold potential for patient stratification and monitoring response to therapy [30,162].

In parallel, therapeutic strategies targeting Wnt signaling are being actively explored. Porcupine inhibitors, such as Wnt974 (LGK974), have been evaluated in early-phase clinical trials for advanced solid tumors, including GI cancers. These agents inhibit Wnt ligand secretion, leading to pathway suppression and the modulation of related biomarkers, such as Axin2. While objective responses were limited, some patients achieved disease stabilization, and early evidence suggests Wnt pathway inhibition may favorably alter the tumor immune microenvironment, supporting future combination strategies with immunotherapies [136]. Similarly, CGX1321, another porcupine inhibitor, demonstrated encouraging activity in patients with RSPO or RNF43-mutated gastrointestinal tumors. Both monotherapy and its combination with pembrolizumab were well tolerated, with manageable side effects. Stable disease was observed in the majority of genetically selected patients, and partial responses were reported in combination-treated cohorts. These findings reinforce the therapeutic relevance of Wnt-targeting agents, particularly in patients with limited response to conventional or immune-based treatments [163].

Recent studies have highlighted the diagnostic and prognostic potential of non-coding RNAs (miRNAs, lncRNAs, and circRNAs) regulating the Wnt/β-catenin pathway in gastrointestinal cancers. These molecules can be detected in liquid biopsies and may reflect real-time pathway activity. For example, miR-148a and miR-148b, which modulate Wnt1/β-catenin signaling, are associated with tumor suppression in HCC. The detection of such ncRNAs in blood-based assays may enable noninvasive patient stratification and disease monitoring, providing new opportunities for early diagnosis and precision oncology [30]. Additionally, it has been demonstrated that fisetin regulates Wnt/β-catenin signaling in colorectal cancer cells by downregulating β-catenin and TCF4, along with their downstream target genes such as cyclin D1 and matrix metalloproteinase 7 (MMP7), thereby attenuating tumor-promoting signaling activity in HT-29 cells [164].

Several Wnt-targeted therapies are currently in Phase 2 clinical trials for GI cancers, and some are also being evaluated in combination with other therapies, such as immunotherapy, and demonstrate great potential for treating patients with GI malignancies.

A growing body of evidence indicates that combination therapies in GI cancers, such as combining target agents with immunotherapy, chemotherapy, or radiotherapy, improve the efficacy and overcome limitations of single-agent treatments. These combinations in treatment strategy aim to leverage the strengths of each modality, targeting different aspects of cancer biology and TME. Targeted therapies are focused on specific molecular pathways involved in tumor development and survival and might be combined with immunotherapy to enhance their effects, such as the immune microenvironment that could be modulated by some targeted agents, leading to immune attack via regulatory T cells (Tregs) and myeloid-derived suppressor cells (MDSCs) on tumor cells. Targeted therapies might also enhance the presentation of tumor antigens to immune cells, improving the priming of tumor-specific T cells as well as targeting EGFR, VEGFR, or BRAF, which are frequently mutated or dysregulated in GI cancers [165]. In addition, it was reported that in patients with GI malignancies, chemotherapy as well as radiotherapy can be combined with immunotherapy to improve synergistic effects. Chemotherapy can also sensitize cancer cells to immune-mediated destruction, because some chemotherapeutic agents can induce immunogenic cell death, releasing tumor antigens that can be recognized by the immune system. Moreover, chemotherapy can modulate the tumor microenvironment, making it more conducive to immune cell infiltration and activity [165]. Radiotherapy may enhance the presentation of these antigens by dendritic cells, leading to a stronger immune response as well as modulating the tumor microenvironment by increasing the expression of immune checkpoint molecules such as PD-L1, potentially making tumors more responsive to immunotherapy. While the combination of radiotherapy and immunotherapy is promising in GI cancers, as it can target both local tumor control and systemic disease. It was reported that targeted therapies and chemotherapy directly destroy malignant cells, whereas immunotherapy may activate the immune system to target and kill tumor cells [166,167].

Numerous findings proved that TME significantly impacts cancer progression and treatment response by influencing cancer cell behavior, metabolic activity, as well as immune cell infiltration. Therefore, the differences in stromal composition with various cell types, such as cancer-associated fibroblasts (CAFs), endothelial cells, and the extracellular matrix (ECM), as well as metabolic context within the TME, induce diverse cancer behaviors, including alteration in growth of tumor, metastasis, and resistance to therapy [168]. It was proven that the TME plays a dual role in cancer development and thus can either promote or inhibit anti-tumor immune response via the function of immune cells (lymphocytes, macrophages, dendritic cells), and other immune cells that reside within the TME. In addition, tumor cells can evade immune surveillance by several pathways, e.g., expressing immune checkpoint molecules (such as PD-L1), resulting in depression of T cell activity or recruiting immunosuppressive cells (such as regulatory T cells). Moreover, the TME is able to induce T cell exhaustion and dysfunction, inhibiting their ability to target and destroy malignant cells. A growing body of evidence reports that TME is characterized by metabolic heterogeneity, with cancer cells and other cells exhibiting different metabolic phenotypes. These modulate their metabolism to adapt to nutrient-poor or hypoxic conditions in the TME. Thus, TME is able to influence the metabolism of immune and stromal cells, potentially promoting tumor growth and metastasis, as well as exceeding other cells for nutrients [169,170]. Additionally, the TME creates metabolic constraints on immune cells, affecting their function and survival [169,170]. Therefore, the TME has a dynamic and complex structure in which tumor cells interact with their surrounding environment, leading to diverse tumor behaviors and responses to therapy. Understanding these interactions is crucial for developing effective cancer therapies [169,170]. 

Particular Wnt ligands and receptors are preferentially dysregulated in certain gastrointestinal (GI) cancers due to a combination of factors, including the type of cancer, the specific genetic mutations involved, and the context of the tumor microenvironment. Mutations in key Wnt pathway components, such as APC and β-catenin, lead to β-catenin accumulation and uncontrolled transcription of Wnt target genes and uncontrolled signaling, while in some mechanisms concerning RNF43 and ZNRF3, E3 ubiquitin ligases can regulate Wnt receptor levels, leading to increased Wnt signaling by preventing receptor degradation. Some mechanisms include crosstalk with other signaling pathways, such as BRAF, EGFR, or BMP signaling, which can impact Wnt pathway activity. Presented dysregulations cause aberrant Wnt/β-catenin signaling, which stimulates growth and the metastasis of GI cells [171]. Therefore, understanding the role of particular Wnt ligands and receptors involved in different cancers is extremely important to explore novel, personalized targeted therapies, and is under extensive examination in clinical trials. Moreover, overcoming resistance and minimizing off-target effects are key challenges for further medicine [134].

There is a growing trend towards intensifying systemic therapies in GI cancers while considering alternatives to invasive surgery. Therefore, further investigations are needed to optimize these therapies and improve their effectiveness and safety. The challenges that have historically limited the clinical success of Wnt-targeting drugs include toxicity, disruption of normal tissue homeostasis, and on-target gastrointestinal side effects. It was proven that the Wnt signaling pathway is crucial for the development of normal tissue as well as homeostasis; thus, disrupting this pathway can lead to toxicity in other tissues. Moreover, Wnt signaling is crucial for maintaining the balance and physiological function of various tissues; therefore, these therapies may disrupt this balance, resulting in adverse effects, e.g., on the gastrointestinal tract and hematopoiesis [68]. Furthermore, the challenges highlight the need for more targeted and specific Wnt inhibitors, as well as strategies to minimize off-target effects and improve drug delivery that are resistant to Wnt-targeted therapies.

Further research is needed to identify more effective and specific Wnt-targeted therapies for GI cancers. Crucial factors that can influence the overall efficacy and toxicity of the combination therapy are the timing, sequence, and dosage of these agents.

## 6. Conclusions

The Wnt/β-catenin signaling pathway is implicated in the development and progression of GI cancers. This highly conserved pathway regulates crucial processes, such as cell proliferation, differentiation, migration, and cancer stem cells. Our review highlights a diverse set of ligands, receptors, co-receptors, and intracellular components within this pathway that show altered expression patterns across different tumor types. The dysregulation of Wnt signaling, often through aberrant expression or mutation of its components, leads to uncontrolled cellular growth and tumorigenesis in gastrointestinal tissues. Its persistent activation has been associated not only with tumor initiation but also with metastasis, treatment resistance, and poor clinical prognosis. Many of these elements analyzed within this pathway demonstrate strong potential as diagnostic, prognostic, or predictive biomarkers.

In GC, overexpression of Wnt ligands (Wnt2, Wnt3, and Wnt5A) and receptors (FZD7 and LRP5/6) is associated with more advanced disease, poorer prognosis, and elevated metastatic potential, while β-catenin and reduced APC expression further underline more aggressive tumor behavior. Interestingly, higher GSK3β levels correlate with better outcomes, highlighting tissue-specific roles. In CRC, aberrant expression of Wnt family members (especially Wnt2, Wnt3A, and Wnt4), FZD receptors, and p-LRP6 promotes EMT and invasiveness. Mutations in APC are a hallmark of CRC, found in over 70% of cases, driving β-catenin accumulation. Nuclear β-catenin localization consistently indicates worse prognosis. EC is characterized by elevated expression of multiple Wnt ligands (notably Wnt2, Wnt3, Wnt5A, and Wnt6) and FZD receptors, often correlating with poorer differentiation, higher potential of tumor invasion, and shorter survival. Loss of Axin and nuclear β-catenin localization further support pathway activation. In HCC, the upregulation of ligands (Wnt1, Wnt3A, etc.) and FZD receptors (FZD2 and FZD7), along with frequent CTNNB1 mutations, is linked to early tumorigenesis, elevated risk of recurrence, and worse clinical outcomes. LRP6 upregulation contributes to therapy resistance, and nuclear β-catenin is common in poorly differentiated tumors. Finally, in PC, overexpression of Wnt ligands (especially Wnt2, Wnt3A, and Wnt7A), FZD family members, and nuclear β-catenin is related to worse prognosis and the presence of distant metastasis. Interestingly, the combined overexpression of PROX1 and β-catenin is associated with improved survival in some patients.

In summary, our analysis indicates that the components of the Wnt/β-catenin signaling pathway hold substantial potential to serve as novel biomarkers in the diagnosis and management of GI cancers. However, further research is needed to understand the complex interplay between these therapies and to optimize combination strategies for different GI cancer subtypes and patient populations. Personalized medicine approaches, where patients are selected for Wnt-targeted therapies based on their tumor’s genetic profile, may improve treatment efficacy.

## Figures and Tables

**Table 1:** Selected key components of the Wnt/β-catenin pathway and their functions.

Component	Location	Function in the Wnt/β-Catenin Pathway	Activation/Inhibition Role	References
DVL	Cytoplasm/membrane	Transmits Wnt signal; recruits AXIN and GSK-3β	Activator	[31,33,34,35]
APC	Cytoplasm	Facilitates β-catenin degradation, preventing pathway activation	Inhibitor	[36]
LRP5/6	Plasma membrane	Co-receptor for Wnt; phosphorylation leads to β-catenin stabilization	Activator	[32,34,37]
RSPO	Extracellular space	Enhances Wnt signaling by inhibiting RNF43/ZNRF3-mediated degradation of FZD receptors	Activator—amplifies Wnt response	[33,38]
Wnt (ligands)	Extracellular space	Initiates signaling by binding to FZD and LRP5/6 receptors	Activator	[38]
FZD	Plasma membrane	Wnt receptor; transduces signal to DVL	Activator	[38]
Axin	Cytoplasm/membrane	Scaffolding protein; assembles β-catenin destruction complex	Inhibitor	[38]
GSK-3β	Cytoplasm	Phosphorylates β-catenin, marking it for degradation	Inhibitor	[38]
CK-1α	Cytoplasm	Phosphorylates β-catenin and LRP6	Dual role—supports inhibition (β-catenin phosphorylation) and activation (LRP6 phosphorylation)	[34,37]
β-catenin	Cytoplasm/nucleus	Transcriptional co-activator; activates Wnt target genes	Activator	[38]
β-TrCP	Cytoplasm	Ubiquitin ligase marking β-catenin for degradation	Inhibitor	[38]
TCF/LEF	Nucleus	Transcription factors activated by β-catenin	Activator	[38]
Groucho/TLE	Nucleus	Repressor of TCF/LEF; inhibits Wnt target gene expression in the absence of β-catenin	Inhibitor—prevents unintended activation	[38]
PORCN	Endoplasmic reticulum/golgi	Enzyme required for Wnt ligand secretion and lipid modification	Activator—essential for Wnt secretion	[38]

APC: adenomatous polyposis coli; Axin: Axin proteins; β-TrCP: beta-Transducin Repeat-Containing Protein; CK1: casein kinase I; DVL: disheveled protein; FZD: specific sevenfold transmembrane receptor Frizzled protein; Groucho/TLE: Drosophila Groucho (Gro) protein/transducin-like enhancer of split; GSK-3β: glycogen synthase kinase-3β; LRP6: phosphorylated lipoprotein receptor 5/6; PORCN: porcupine O-acyltransferase; RNF43/ZNRF3: Ring Finger Protein 43/Zinc and Ring finger 3; RSPO: R-spondins; TCF/LEF: β-catenin-T-cell factor/lymphoid enhancer-binding factor; Wnt: wingless-type MMTV integration site family.

**Table 2:** Summary of the Wnt/β-catenin pathway components as biomarkers in GI cancers.

GI Cancer	Pathway Component	Biomarker Relevance	Clinical Significance	References
Gastric Cancer	Wnt2	Prognostic	Overexpression was linked to advanced stage and lymph node metastasis	[83]
	Wnt3	Therapeutic target	Silencing was shown to reduce proliferation, promote apoptosis	[84]
	Wnt5A	Prognostic	Expression was correlated with invasion, metastasis, and poor survival	[86,87,88]
	FZD7	Prognostic/predictive	High expression was linked to metastasis and immune response	[90]
	LRP5/LRP6	Prognostic/therapeutic target	High expression was associated with advanced stage and cancer stemness	[94,95]
	β-catenin	Prognostic	High expression was correlated with poor differentiation and metastasis	[97,98]
	APC	Prognostic	Expression was downregulated via miRNAs and linked to increased proliferation	[99]
	GSK3β	Prognostic	Higher expression was observed in early stages and associated with better survival	[100]
Colorectal Cancer	Wnt2/Wnt3A/Wnt4	Diagnostic/prognostic	Expression was associated with EMT, invasion, and angiogenesis	[103,104,105]
	Wnt5A	Prognostic	Higher expression was correlated with better prognosis	[106]
	FZD6/FZD7	Prognostic	Elevated expression was found in tumor core and linked to Wnt activation	[107,108]
	p-LRP6	Prognostic	Expression correlated with advanced stage and shorter DFS	[109]
	β-catenin	Prognostic	Nuclear β-catenin staining in tumor cells has been associated with poor prognosis; The presence of B-catenin mutations could help to identify individuals at higher risk of developing CRC and guide personalized treatment strategies	[110,111,112,113,114]
	APC	Diagnostic/prognostic	The presence of APC mutations could help to identify individuals at higher risk of developing CRC and guide personalized treatment strategies	[113,114]
	GSK3β/CK1	Prognostic/therapeutic target	Elevated levels were linked to chemoresistance and poor survival	[116,117]
Esophageal Cancer	Wnt2/Wnt3/Wnt5A/Wnt6	Prognostic/therapeutic target	High expression was linked to EMT, metastasis, and shorter OS	[99,119,120,121]
	FZD2/FZD6/FZD7	Prognostic	Overexpression was correlated with invasion and poor PFS	[119,123,124]
	LRP6	Prognostic/therapeutic	Expression promoted cell proliferation and was associated with poor differentiation	[125]
	Axin	Prognostic	Low expression was linked to invasion and lymph node metastasis	[128,136]
	β-catenin	Diagnostic/prognostic	The presence of B-catenin mutations could help to identify individuals at higher risk of developing EC; β-catenin mutations have been associated with tumor aggressiveness and metastasis, potentially aiding in predicting disease progression	[30,130,131]
Liver Cancer (HCC)	Wnt1/Wnt3A/Wnt5A/Wnt10B	Prognostic	Overexpression was correlated with poor survival and recurrence	[137,138,139]
	FZD2/FZD7	Prognostic/therapeutic target	Expression was linked to EMT and early tumorigenesis	[140,141]
	LRP6	Prognostic/therapeutic target	Upregulation was associated with drug resistance and poor prognosis	[142]
	β-catenin	Prognostic	Nuclear localization was linked to poorly differentiated HCC	[143]
	CTNNB1 mutations	Diagnostic	Mutations were found in ~20–40% of HCC cases, especially those related to HCV	[144,145]
	Axin1/2/GSK3β/APC	Prognostic	Mutations or suppression were found to drive pathway activation	[146,147]
Pancreatic Cancer (PDAC/PAAD)	Wnt2/Wnt3A/Wnt5A/Wnt7A	Prognostic/therapeutic target	Expression was linked to metastasis and poor prognosis	[128,150,151,152,153]
	FZD1–FZD10	Prognostic/therapeutic target	Overexpression was correlated with disease progression and clinical stage	[154]
	β-catenin	Prognostic	High expression was linked to better survival in some contexts	[155]
	PROX1 + β-catenin	Prognostic	Combined expression was indicative of improved survival	[156]
	GSK3β/Axin/APC	Functional regulators	Impaired β-catenin degradation was linked to its accumulation	[155]

APC: adenomatous polyposis coli; Axin: Axin proteins; β-TrCP: beta-Transducin Repeat-Containing Protein; CK1: casein kinase I; CRC; colorectal cancer; CTNMB1: Catenin Beta 1 gen; DFS: disease-free survival, DVL: disheveled protein; EMT: epithelial-to-mesenchymal transition; FZD: specific sevenfold transmembrane receptor Frizzled protein; GC: gastric cancer; Groucho/TLE: Drosophila Groucho (Gro) protein/transducin-like enhancer of split; GSK-3β: glycogen synthase kinase-3β; HCC: hepatocellular carcinoma; HCV: hepatitis C virus; LRP6: low-density phosphorylated lipoprotein receptor 5/6; OS: overall survival; PFS: progression-free survival; pLPR: phospho low-density lipoprotein receptor-related protein; PORCN: porcupine homolog–Drosophila human gene; RNF43/ZNRF3: Ring Finger Protein 43/Zinc and Ring finger 3; PROX1: prospero homeobox protein 1; RSPO: R-spondins; TCF/LEF: β-catenin-T-cell factor/lymphoid enhancer-binding factor; Wnt: wingless-type MMTV integration site family.
